# Effects of Enzymatic Liquefaction, Drying Techniques, and Wall Materials on the Physicochemical Properties, Bioactivities, and Morphologies of Zinc-Amaranth (*Amaranthus viridis* L.) Powders

**DOI:** 10.1155/2021/1819104

**Published:** 2021-10-21

**Authors:** Siti Faridah Mohd Amin, Roselina Karim, Yus Aniza Yusof, Kharidah Muhammad

**Affiliations:** ^1^Faculty of Food Science and Nutrition, Universiti Malaysia Sabah, 88400 Kota Kinabalu, Sabah, Malaysia; ^2^Faculty of Food Science and Technology, Universiti Putra Malaysia, 43400 UPM Serdang, Selangor Darul Ehsan, Malaysia; ^3^Faculty of Engineering, Universiti Putra Malaysia, 43400 UPM Serdang, Selangor Darul Ehsan, Malaysia

## Abstract

The demand for vegetable powder has been escalating considerably due to its various health benefits and higher shelf life compared to fresh green leafy vegetables. Thus, much research emphasised manufacturing vegetable powder at a lower operational cost and higher efficiency while preserving the nutritive values of the vegetables. In this study, zinc- (Zn-) amaranth puree was liquefied with three types of cell wall degrading enzymes (i.e., Viscozyme L, Pectinex Ultra SP-L, and Rapidase PAC) with varying concentrations (0–3% v/w) and incubation time (0.5–24 h) at pH 5 and 45°C before the drying process. The results showed that enzymatic liquefaction using 1% (v/w) of Viscozyme L for 3 h was the optimal procedure for the reduction of the viscosity of the puree. The liquefied puree was then microencapsulated through either spray- or freeze-drying with different wall materials, e.g., 10% of maltodextrin (MD) DE 10, resistant maltodextrin (RMD), N-octenyl succinate anhydride (OSA) starches from waxy maize, HI CAP 100 (HICAP), Capsul (CAP), and gum Arabic (GA). The results showed that all freeze-dried powders generally had higher process yield (except for that encapsulated by HICAP), higher moisture content (but similar water activities), higher retention of total Zn-chlorophyll derivatives, lower hygroscopicity with slab-like particles, larger particle size, and lower bulk density than those of spray-dried powders. In contrast, the spray-dried powders exhibited irregular spherical shapes with relatively high encapsulation efficiency and antioxidant activities. Nonetheless, encapsulation using different wall materials and drying methods had no significant effect on the powder's cohesiveness and flowability.

## 1. Introduction

Green amaranth or Chinese spinach (*Amaranthus viridis*), commonly known as *bayam hijau* or *bayam panjang* in Malay, is one of the most popular leafy vegetables consumed in Malaysia. Aside from *A. viridis*, there are also other species of amaranth that are abundantly available in the market, namely, *bayam itik* (*Amaranthus blitum*), *bayam merah* (*Amaranthus gangeticus*), and *bayam putih* (*Amaranthus paniculatus*) [1]. *A. viridis* has both round and tapered leaves, while the latter was used in this study. Previous studies showed that fresh amaranth leaves have higher chlorophyll content than other local vegetables such as lettuce, water spinach, and broccoli, therefore exhibiting higher antioxidant activity [2]. However, the chlorophyll components in green amaranth have the propensity to degrade during food processing into puree and bioactive component extraction due to heat, acidification, and upon contact with oxygen or light [3, 4]. Thereupon, to surpass these adverse conditions, the formation of stable chlorophyll molecules in green amaranth was initiated by substituting magnesium ion (Mg) with Zn in the porphyrin ring of Mg-free chlorophyll derivatives, such as pyropheophytins or pheophytin [6]. This approach allows the Zn-chlorophyll derivatives to have a similar colour to Mg-chlorophyll while being more stable and thermally resistant in low-pH food [7].

Aside from the issue that arose from the biochemical components of the plant, the fresh amaranth and its puree have a short shelf life due to moisture-driven deterioration [8]. Moreover, there are several limitations to the commercial-scale application of the puree form [9]. Therefore, the production of amaranth powder has been an effective approach being taken to enhance the functionality, versatility in applications and retention of bioactive compounds within the powder as well as to extend its shelf life [12, 13]. In addition, the demand for fruit and vegetable powders has been increased recently owing to the multiple health benefits of the application of these powders in varieties of food formulations [14]. The powder can be formed through many drying methods, one of which is spray-drying.

Feed mixture viscosity is one of the key parameters in spray-drying [10], which affects the ease of pumping and atomisation of the feed mixture into the spray-dryer to achieve an appropriate powder yield [5]. The high viscosity of the amaranth puree, however, can be difficult to pump and atomise in a spray-drying operation [5]. Thus, enzymatic liquefaction of amaranth is a feasible solution to reduce the viscosity in spray-drying [11], which can be achieved by using enzymes that specifically break down the individual types of polysaccharides in the cell wall structure of amaranth.

Apart from reducing the viscosity of amaranth puree, the encapsulation technique and wall material being used should be selected accordingly to maximise the functionality and retention of compounds within the microcapsules of the amaranth powder, as well as reducing the operational cost [15, 18]. The wall material acts as a coating material or barrier to isolate the core from the outside environment, which determines the physicochemical, biological, and morphological properties of the microcapsules [9]. There are different types of encapsulating agents, such as maltodextrin (MD) and gum Arabic (GA), which are commonly used in spray-drying due to their low viscosity and high solubility [16]. Alternatively, resistant maltodextrin (RMD), HI CAP 100 (HICAP), and Capsul (CAP) exhibited strong emulsifying, oxygen-impermeable, and wall-forming properties to provide stability against environmental stress (e.g., pH, temperature, and ionic strength) [17].

Several drying technologies of encapsulation have been established, depending on the type of core and wall materials, size of the capsules, resistivity to high temperature, and physical state of the starting materials [19]. Presently, spray-drying, freeze-drying, vacuum-drying, spray-cooling, liposome entrapment, extrusion, cocrystallisation, and emulsion are among some of the preferred encapsulation techniques espoused in the food industry [20, 21]. Nonetheless, spray- and freeze-drying are the most broadly adopted encapsulation techniques [19, 22]. The main advantages of the spray-drying process are high powder yield and reduced time of exposure to high temperature, resulting in reduced thermal damage of the core materials. According to Hammami and René [23], this technique is at least four times more economical than freeze- and vacuum-drying due to its lower electricity consumption and shortened drying time [24].

Several studies have been conducted to compare the influence of different drying methods and encapsulating agents on the physicochemical properties of fruit and vegetable powder, including red beetroot [25], blueberry [26], Aronia berry [27], and acerola cherry [28]. Nevertheless, the studies on the effects of spray- and freeze-drying using different wall materials on enzyme-liquefied amaranth puree were still lacking. In this regard, the objective of this study is to evaluate the physicochemical properties, bioactivity, and morphology of microcapsules produced from spray- and freeze-drying of enzyme-liquefied Zn-amaranth puree. Briefly, the fresh green amaranth puree was prepared through mechanically grinding. An enzyme used for the liquefaction of the puree was then selected among Viscozyme L, Pectinex Ultra SP-L, and Rapidase PAC after several optimisation steps. The enzyme-liquified puree was then mixed with different wall materials (i.e., MD, RMD, HICAP, CAP, and GA) and subjected to spray- and freeze-drying. After which, physicochemical analyses were conducted to evaluate the effectiveness of the drying technique, based upon their process yield, moisture content, water activity, hygroscopicity, encapsulation efficiency, particle size distribution, powder density, Hausner ratio, and Carr's index. The biological activity of the powder was elucidated by two free radical scavenging assays. In addition, the total Zn-chlorophyll derivatives of Zn-amaranth puree and Zn-chlorophyll derivative retention of powder were determined. Lastly, the morphology of the powder was examined using a scanning electron microscope.

## 2. Materials and Methods

### 2.1. Materials

Fresh green amaranth (*A. viridis* L.) with tapered leaves was purchased from a local market in Kuala Lumpur, Malaysia. Zinc chloride (ZnCl_2_), acetone, 2,2-diphenyl-1-picrylhydrazyl (DPPH), and 6-hydroxy-2,5,7,8-tetramethylchroman-2-carboxylic acid (Trolox) were procured from Sigma-Aldrich (St. Louis, MO, USA). 2,4,6-Tri(2-pyridyl)-s-triazine (TPTZ) was obtained from Fluka (Germany). Methanol, iron (III) chloride hexahydrate (FeCl_3_.6H_2_O), and hydrochloric acid (HCl) were received from Merck (Darmstadt, Germany). Sodium hydroxide (NaOH) was purchased from R&M Chemicals (Essex, UK). Viscozyme L (≥100 FBG/g) and Pectinex Ultra SP-L (≥3800 units/mL) were purchased from Novozymes Inc. (Copenhagen, Denmark). Rapidase PAC (≥95000 AVJP/g) was kindly supplied by DSM Food Specialties (Delft, Netherlands). The main characteristics of the enzymes are summarised in Table 1. Encapsulating agents and maltodextrin DE 10 (MD) were obtained from San Soon Seng Food Industry, Malaysia, and resistant maltodextrin (Fibresol-2, RMD) was purchased from ADM Company, USA. Gum Arabic (GA) was purchased from R&M Chemicals (Essex, UK). N-octenyl succinate anhydride (OSA) starches, viz., Capsul (CAP) and HI CAP 100 (HICAP), were purchased from Ingredion Malaysia Sdn. Bhd, Malaysia.

### 2.2. Preparation of Zn-Amaranth Puree

Fresh amaranths were washed, drained, chopped, and removed from their roots. Thereafter, the leaves with stalks were blended in a Waring blender for 5 min at high speed to obtain the amaranth puree. After which, the puree was treated at pH 8 (adjusted with citric acid or NaOH) and 90°C for 15 min with 1500 mg/L of ZnCl_2_ [29]. The Zn-amaranth puree was then stored at –20°C and thawed at 4°C before the liquefaction process.

### 2.3. Enzymatic Liquefaction of Zn-Amaranth Puree

The selection of suitable enzyme, optimisation of the enzymatic reaction, and investigation of the effects of enzymatic liquefaction on the viscosity of Zn-amaranth puree consisted of three parts. The pH and temperature for the liquefaction process were optimised based on parameters given by the manufacturers (Table 1). Firstly, the effects of Viscozyme L, Rapidase PAC, and Pectinex Ultra SP-L on the viscosity and Zn-chlorophyll derivative contents of puree were screened at 1–3% (v/w) of enzyme concentration at pH 5 and 45°C for 24 h.

The pH of the mixture was adjusted with 1% (v/v) citric acid and 1% NaOH solution, whenever necessary. At the end of the incubation, the enzyme was inactivated by heating the reaction mixture at 90°C for 5 min [30]. The viscosity of liquefied Zn-amaranth puree was measured at room temperature (24 ± 1°C) using a rheometer (Anton Paar Physica, Gmbh, Germany) at 160 rpm with a spindle no. 3. An aliquot of the liquefied puree was filled into a measuring cup (52.6 mm in diameter, 75 mm in height, and 150 mL in volume), and its viscosity was expressed in millipascal second (mPa.s). The enzyme with the highest activity, which produced the lowest viscosity of the puree, was then selected for the subsequent assays. Secondly, the optimum enzyme concentration was identified by repeating the previous assay while using a smaller scale of enzyme concentration (0, 0.25, 0.5… 3% v/w). Lastly, the incubation time was then fine-tuned by repeating the assay with the enzyme and its optimum concentration identified from the previous section, while varying the incubation time (0.5–24 h). Thereafter, the spray- and freeze-drying of the puree were performed by using the identified enzyme with its optimised concentration and incubation time.

### 2.4. Preparation of Microencapsulated Zn-Amaranth Powder

The previously liquefied Zn-amaranth puree was first sieved through a plastic nylon strainer with a fine sieve of 0.4 mm mesh size to remove undigested coarse fibres that might clog the spray-drying atomiser nozzle. Next, the puree (10% (w/w)) was mixed with different wall materials (MD, RMD, HICAP, CAP, or GA) and was homogenised for 10 min using a Waring blender at high speed, followed by filtering through a 150 *μ*m mesh filter screen. Half of the mixture was spray-dried using a pilot-scale spray drier (Niro A/S, GEA, Germany) at inlet and outlet temperatures of 150 and 85°C, respectively, with a feed rate of 10 mL/min and a rotary atomiser with a speed of 15000 rpm. Another half of the mixture was frozen at –20 ± 1°C for 24 h and dried in a freeze dryer (Labconco, USA) at –50°C for three days until completely dried. Subsequently, all dried mixtures were ground to powder, filled in an aluminium laminated polyethene pouch, sealed, and stored at 4°C until further analysis.

### 2.5. Physicochemical Analyses of Spray- and Freeze-Dried Zn-Amaranth Powders

#### 2.5.1. Process Yield

The process yield of the spray- and freeze-dried Zn-amaranth powder was calculated based on the relationship between the total solid (TS) content in the resulting powder and feed mixture using Equations (1) and (2) [10], in terms of the weight percentage of the amount of water loss during the drying process [33].

(1) $$\begin{array}{c} \text{TS content }\left(\%\right)=100-\text{moisture }\left(\%\right), \end{array}$$

(2) $$\begin{array}{c} \text{Process yield }\left(\%\right)=\frac{\text{TS }\left(\%\right)\text{ powder}\times \text{powder yield after drying }\left(\text{g}\right)}{\text{TS }\left(\%\right)\text{ feed mixture}\times \text{feed mixture }\left(\text{g}\right)}\times 100\%. \end{array}$$

#### 2.5.2. Moisture Content and Water Activity (*a*_*w*_)

The moisture content of Zn-amaranth powder was determined by oven-drying according to Bhusari et al. [31]. Triplicate of amaranth powder (1 g) were weighed, dried at 105°C for 24 h, and then cooled to ambient temperature in a desiccator until a constant weight was achieved. The water activity (*a*_*w*_) of the powder was determined using a digital water activity meter (Model 3TE, Aqualab, WA).

#### 2.5.3. Percentage of Hygroscopicity

Hygroscopicity of the powder was determined according to Cai and Corke [32] with some modifications. The powder (approximately 1 g) was placed in a desiccator containing a saturated NaCl solution (relative humidity, RH = 75.3%). The results were expressed as gram (g) of absorbed moisture per 100 g dry solids (g/100 g) after seven days of storage.

#### 2.5.4. Encapsulation Efficiency (EE)

The EE was calculated as outlined by Idham et al. [34] with some proposed modifications. To determine the total Zn-chlorophyll derivatives (TZCDs), 100 mg of powder was weighed into a centrifuge tube, followed by the addition of approximately 1 mL of distilled water, and the mixture was then vortexed for 10 min to destroy the microcapsule membrane. Next, 9 mL of 100% acetone was added to the mixture, vortexed, and then centrifuged at 3000 × g for 10 min. Simultaneously, the surface Zn-chlorophyll derivatives (SZCDs) were determined by directly extracting 100 mg of powder with 10 mL acetone, vortexed for 10 min, and then centrifuged at 3000 × g for 10 min. Subsequently, EE was calculated according to Equation (3), which was modified by Barbosa et al. [35] as shown below:

(3) $$\begin{array}{c} \text{EE }\left(\%\right)=\frac{\left(\text{TMCD}-\text{SMCD}\right)}{\text{TMCD}}\times 100\%. \end{array}$$

#### 2.5.5. Particle Size Distribution

The average particle size distribution of spray- and freeze-dried Zn-amaranth powder were measured using a particle size analyser (Mastersizer 2000, Malvern Instruments, UK) following the methodology described by Ng Lay Tze et al. [36], and the result was reported as volume-weighted mean diameter (*D*, *μ*m) [3, 4].

#### 2.5.6. Bulk Density, Hausner Ratio, and Carr's Index

For bulk density (BD) determination, a standard volume of measuring cylinder (10 mL), was used. The initial weight of the empty measuring cylinder and the weight of the powder taken inside the measuring cylinder were recorded. For tapped density (TD), the measuring cylinder was mechanically tapped, and its volume was recorded upon reaching the constant volume. BD, TD, Hausner ratio (HR), and Carr's index (CI) of the powder were determined according to Equations (4)–(7) [36].

(4) $$\begin{array}{c} \text{BD}=\frac{\text{Mass of powder }\left(\text{g}\right)}{\text{Volume of powder }\left({\text{cm}}^{3}\right)}\times 100, \end{array}$$

(5) $$\begin{array}{c} \text{TD}=\frac{\text{Mass of powder }\left(\text{g}\right)}{\text{Final tapped volume }\left({\text{cm}}^{3}\right)}, \end{array}$$

(6) $$\begin{array}{c} \text{HR}=\frac{\text{TD}}{\text{BD}}, \end{array}$$

(7) $$\begin{array}{c} \text{CI}=\frac{\text{TD}-\text{BD}}{\text{BD}}\times 100. \end{array}$$

#### 2.5.7. Preparation of Zn-Amaranth Powder Extract for Antioxidant Assays

Zn-amaranth powder (0.5 g) was diluted in 5 mL of distilled water and vortexed for 15 min until fully dissolved. The mixture was centrifuged at 3000 × g for 10 min, and the supernatant was extracted with 80% methanol (20 mL) at 40°C for 24 h. Upon cooling down to room temperature (27 ± 2°C), the mixture was centrifuged at 3500 × g for 15 min, and the supernatant (extract) was obtained. The extract was added to an airtight glass vial and stored in a refrigerator until further analyses [37].

#### 2.5.8. 2,2-Diphenyl-1-Picrylhydrazyl (DPPH) Assay

The antioxidant activity of the extract was measured using the DPPH assay as outlined by Brand-Williams et al. [38] with slight modification. Briefly, 3.9 mL of 0.1 mM DPPH solution (prepared using 80% v/v methanol) was mixed with 0.1 mL of sample extract in a test tube. The solution in the test tube was then vortexed for 15 s and incubated in the dark for 15 min at room temperature. The absorbance of the solution in the test tube was measured using a UV-Vis spectrophotometer (Shimadzu, Japan) at 515 nm with 80% methanol (v/v) as the blank. The assay was carried out in triplicate. Trolox was used for the preparation of the standard curve, and the results were expressed in mM of Trolox Equivalents (TE).

#### 2.5.9. Ferric Reducing Antioxidant Power (FRAP) Assay

The FRAP assay was carried out by adopting the procedure proposed by Benzie and Strain [39] with minor modifications. Briefly, the FRAP reagent was prepared by mixing sodium acetate buffer (300 mM, pH 3.6), 10 mM of 2,4,6-tripyridyl-s-triazine (TPTZ) solution in 40 mM of HCl, and 20 mM of FeCl_3_ solution in the proportion of 10 : 1 : 1 (v/v), respectively. The FRAP reagent was freshly prepared and was warmed to 37°C in a water bath before use. Thereafter, the extract (150 *μ*L) was added to the FRAP reagent (2.85 mL), and the reaction mixture was allowed to incubate for 30 min at room temperature. The absorbance of the reaction mixture was recorded at 593 nm using a UV-Vis spectrophotometer (Shimadzu, Japan). The assay was carried out in triplicate. A standard curve was constructed using Trolox, and the results were expressed in mM of TE.

#### 2.5.10. Extraction and Determination of Total Zn-Chlorophyll Derivatives (TZCDs) of Zn-Amaranth Puree and Zn-Chlorophyll Derivative Retention of Powder

The measurement of the Zn-chlorophyll derivative content was performed according to Dere et al. [40] with minor modifications. The extraction of the Zn-chlorophyll derivatives from the liquefied Zn-amaranth puree was carried out in dim light using a stoppered tube covered with aluminium foil to reduce the photo destruction of the samples. The fresh amaranth (1 g) was ground with 100% acetone using a mortar and pestle until the residue became colourless. The content of the mortar was then transferred into a centrifuge tube by rinsing with 100% acetone several times at the maximum of 50 mL. Thereafter, the mixture was centrifuged at 3500 × g for 10 min, and the supernatant was collected and diluted up to 50 mL with 100% acetone and centrifuged again at 3500 × g for 10 min, while for the spray- or freeze-dried powder, about 0.5 g of powder was diluted in 10 mL of distilled water and vortexed for 15 min until the powder fully dissolved. The extract of the powder was then centrifuged (3000 × g) for 10 min, and the supernatant was transferred into a centrifuge tube. The supernatant was then diluted up to 50 mL with 100% acetone and centrifuged at 3500 × g for 10 min. Subsequently, the absorbance of the extracted Zn-chlorophyll derivatives in the supernatant obtained from fresh amaranth and powder was measured at 662 and 645 nm against 100% acetone as the blank using a UV-Vis spectrophotometer (Shimadzu, Japan). Equations (8) and (9) were used for the quantification of the TZCD (mg/g fresh weight, FW) and the TZCD retention [41].

(8) $$\begin{array}{c} \text{TZCD}=\frac{\left(16.26A_{645}+7.79A_{662}\right)\times \text{dilution factor}}{1000}, \end{array}$$

(9) $$\begin{array}{c} \text{TZCD retention }\left(\%\right)=\frac{\text{ total Zn}-\text{chlorophyll derivatives}\left(\text{mg}/\text{g}\right)\times \left(100-\text{moisture feed}\right)}{\text{total Zn}-\text{chlorophyll derivatives }\left(\text{mg}/\text{g}\right)\times \left(100-\text{moisture powder}\right)}\times 100\%, \end{array}$$

where *A*_645_ and *A*_662_ are the absorbances at 645 and 662 nm, respectively.

#### 2.5.11. Morphology of Spray- and Freeze-Dried Powders

The observation of the morphology of spray- and freeze-dried Zn-amaranth powder was conducted using a scanning electron microscope (SEM) (LEICA Electron Microscopy Ltd., Cambridge, England). The powder was attached to a metallic stub with double-sided adhesive tape and coated with a thin layer of gold (60 nm) in a vacuum evaporator. The SEM was operated at 20 kV with a magnification of ×200.

### 2.6. Statistical Analysis

The data acquired were expressed as the means ± standard deviations of three replicates. The MINITAB (version 16) statistical software was employed for one-way analysis of variance (ANOVA) and Tukey's test for the determination of the significant differences between the means of triplicate at the 5% level.

## 3. Results and Discussion

### 3.1. Selection of Enzymes for Liquefaction of Zn-Amaranth Puree

**Figure 1:**
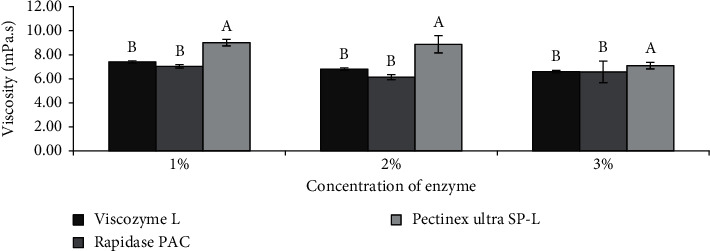
Effects of various types of cell wall degrading enzyme concentrations on viscosity (mPa.s) of Zn-amaranth puree at pH 5 and 45°C after 24 h. Data are expressed as the means ± standard deviation of triplicates. Means with the same letter in each column mean not significantly different (*p* < 0.05, ANOVA).

Although the viscosity of the puree can be regulated by adding water, a study by Chong and Wong [43] reported that liquefaction using enzymes with cellulolytic, hemicellulolytic, and pectinolytic activities is a better method, as the former could result in higher energy consumption for the removal of the additional water during spray-drying [9]. Therefore, three types of commercial cell wall degrading enzymes (i.e., Viscozyme L, Rapidase PAC, and Pectinex Ultra SP-L) at 1, 2, and 3% (v/w) were screened to determine their effects on the viscosity of puree at the experimental condition of pH 5 and 45°C after 24 h. As shown in Figure 1, all purees had a viscosity below 250 mPa.s, which is favourable for further spray-drying as the yield of amaranth juice would be maximised while preventing the spray-dryer nozzles from clogging [42]. Specifically, the purees liquefied with Viscozyme L and Rapidase PAC exhibited lower viscosity than that of Pectinex Ultra SP-L, probably due to the synergistic effects of cellulolytic, pectinolytic, and hemicellulolytic enzymes in degrading the cell wall [44]. Such effects resulted in a reduction of the capacity to retain water and thereby releasing free water into the system which produced low viscosity [45, 46]. This finding is consistent with that of Stoll et al. [47], which stated that carrot liquefaction was the most effective when using a mixture of enzymes with cellulolytic, pectinolytic, and hemicellulolytic activities, as seen from a drastic reduction of viscosity to 21% of the initial value within 90 min at 50°C.

**Figure 2:**
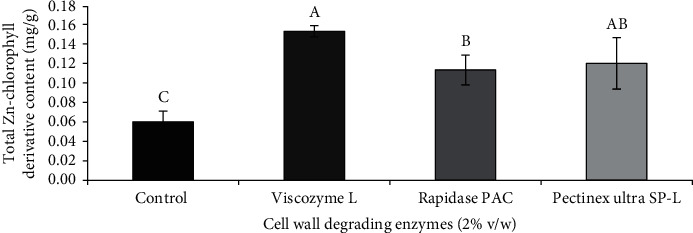
TZCD (mg/g FW) content of Zn-amaranth puree liquefied with 2% of different cell wall degrading enzymes (% v/w) and incubated at pH 5 and 45°C for 24 h. Data are expressed as the means ± standard deviations of triplicates. Means with the same letter in each column mean not significantly different (*p* < 0.05, ANOVA).

The effects of cell wall degrading enzymes on the TZCD content of Zn-amaranth puree were further investigated at a constant condition (2% enzyme concentration at pH 5 and 45°C) after 24 h, and the results are shown in Figure 2. The control (nonenzymatically liquefied puree) had the lowest TZCD content of 0.06 mg/g FW, while that treated with Viscozyme L yielded the highest TZCD content (0.15 mg/g FW), followed by Pectinex Ultra SP-L and Rapidase PAC (0.12 mg/g FW). Thus, Viscozyme L was then chosen for enzymatic liquefaction of the puree.

### 3.2. Effect of Enzyme Concentration on the Viscosity of Liquefied Zn-Amaranth Puree

**Figure 3:**
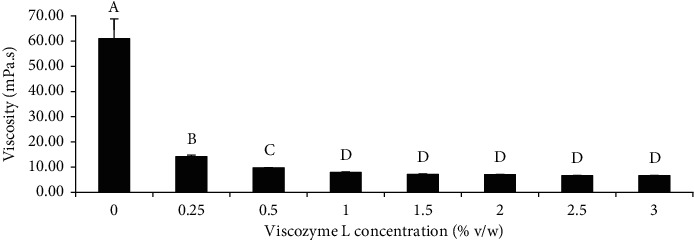
Viscosity of Zn-amaranth puree liquefied at pH 5 and 45°C for 24 h with different concentrations (0–3% v/w) of Viscozyme L. Data are expressed as the means ± standard deviations of triplicates. Means with the same letter in each column mean not significantly different (*p* < 0.05, ANOVA).

After selecting the suitable enzyme, the enzyme kinetic experiment was repeated using a smaller range of Viscozyme L concentration to fine-tune the enzyme consumption for the reduction of manufacturing cost [43].  Figure 3 represents the viscosity of the puree liquefied with different Viscozyme L concentrations (0–3%) and incubated at pH 5 and 45°C for 24 h. There was a significant decrease (*p* < 0.05) in the viscosity of the puree when the enzyme concentration increased from 0 to 1%, while no further significant impact on the viscosity beyond 1%, indicating that 1% of Viscozyme L is optimum for cell wall hydrolysis of the amaranth puree.

### 3.3. Effect of Incubation Time on the Viscosity of Liquefied Zn-Amaranth Puree

**Figure 4:**
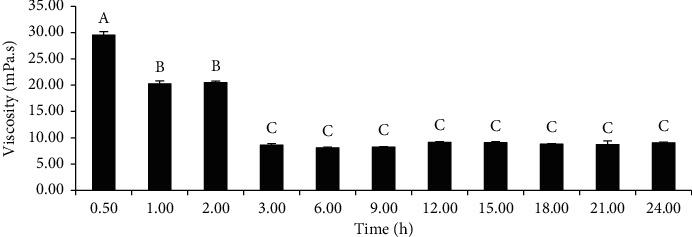
Effect of incubation time (h) on the viscosity (mPa.s) of Zn-amaranth puree liquefied with 1% (v/w) Viscozyme L at pH 5 and 45°C. Data are expressed as the means ± standard deviations of triplicates. Means with the same letter in each column mean not significantly different (*p* < 0.05, ANOVA).

Figure 4 represents the viscosity of Zn-amaranth puree liquefied with 1% (v/w) of Viscozyme L at pH 5 and 45°C for 0.5–24 h. It was observed that the liquefaction of the puree significantly (*p* < 0.05) reduced its viscosity in a time-dependent manner until the maximum of 3 h of incubation. According to Umsza-Guez et al. [48], it is undesirable to liquefy ambarella (*Spondias cytherea* Sonn.) puree at above 90 min of incubation, as it could cause a destructive effect on the operation of the enzymes and release an unfavourable cooked off-flavour. Altogether, the spray- and freeze-drying of Zn-amaranth puree were conducted by incubating it with Viscozyme L (1% v/w) at pH 5 and 45°C for 3 h.

### 3.4. Physicochemical Properties of Spray- and Freeze-Dried Zn-Amaranth Powder Coated with Various Wall Materials

All spray-dried powder exhibited moisture content below 5% (Table 2), and the results were in accordance with that of spray-dried acai [10], jussara [49], and grape [49] powder, in which such a low moisture content could have high storage stability [50, 51]. Conversely, the moisture content of freeze-dried powder was found to be higher than that of spray-dried. Specifically, the freeze-dried powder produced with GA showed the highest moisture content (8.38%), probably due to its highest number of hydrophilic groups, therefore leading to the highest adsorption of moisture from the ambient air [52, 53]. Additionally, since freeze-drying is conducted at less than –40°C to enable rapid freezing, the pores in the outer layer are shrunk and smaller than those of spray-dried powder. This phenomenon hinders mass transfer between the internal and external environment, and the outer layer acts as a barrier against sublimation, leading to an increase in moisture retention. In contrast, spray-drying gives lower moisture content than freeze-drying owing to the higher temperature of the former [54, 55]. Moreover, all spray- and freeze-dried powders encapsulated with different wall materials presented *a*_*w*_ values less than 0.6, indicating that the powder was microbiologically stable. Hence, if any spoilage occurred, it is possibly induced by chemical reactions rather than by microorganisms [56].

All powders had hygroscopicity lower than 20%, indicating that they are less hygroscopic (Table 2) [57]. The freeze- and spray-dried powders encapsulated with CAP had the lowest hygroscopicity, suggesting that it could be attributed to the low polarity of the wall material to form hydrogen bonds with water [58]. All freeze-dried powders demonstrated lower hygroscopicity compared to spray-dried powders, probably due to the larger particle size of the former [60], in which the exposed surface area is smaller and consequently leading to a lower water absorption [61]. According to the findings reported by Kuck and Noreña [49] and Saikia et al. [59] which involved grape and star fruit pomace, a lower hygroscopicity can facilitate the conservation and preservation of bioactive compounds [30].

In general, the process yield of all encapsulated powders was higher than 60%, with the yield of freeze-dried powders being higher than that of spray-dried powders (Table 2). A lower yield could be due to some of the spray-dried powder being stuck to the drying chamber and cyclone walls [62], or they were blown out by air and remained on the filter [23]. Such instances were not visible to freeze-drying microencapsulation since there was no drying material flowing in the chamber [63]. Specifically, the freeze-dried powder prepared from MD exhibited the highest yield (96.95%), while that encapsulated with HICAP had the lowest yield (66.35%), probably due to some amount of this powder being lost during the freeze-drying or grinding process.

Encapsulation efficiency (EE) is the potential of an encapsulating agent to hold or encapsulate the core component inside the microcapsule [34]. For instance, a low EE may result in low stability of the core materials as there is no protective effect offered by the capsules against different storage conditions [64]. Hence, an effective encapsulation process may have a high retention of Zn-chlorophyll derivatives and a minimum amount of them on the surface of the powder particles. As demonstrated in Table 2, spray-drying encapsulated Zn-chlorophyll in a manner better than freeze-drying. Our findings were in line with those reported by Samakradhamrongthai et al. [65], in which the EE of spray-dried white champaca (*Michelia alba*) was higher than that of the freeze-dried powder using octenyl succinic anhydride starch (OSA) as an encapsulating agent. It could be due to the droplet-to-droplet interaction in the emulsion during the freeze-drying process, which consumes more time than spray-drying, and therefore resulting in the inconsistency of extract entrapment in the freeze-dried powder [66]. The spray-dried powder encapsulated with MD (91.78%), RMD (90.58%), and GA (90.44%) produced a relatively high EE as compared with others (Table 2), suggesting that EE mainly depends on the wall material composition and type of drying process.

As indicated in Table 3, all freeze-dried powders exhibited a larger particle size than that of spray-dried powders, probably due to low temperature during freeze-drying, which caused a lack of strength to break the frozen particles or to change the surface [67]. Thereupon, the larger particle size of freeze-dried powders may have contributed to the higher chlorophyll derivative retention compared to spray-dried powders. Gong et al. [68] stated that powder with a small particle size (<50 *μ*m) resulted in handling difficulties and poor reconstitution properties. Moreover, powder with a small particle size (and therefore large surface area) is vulnerable to various environmental factors, thus increasing the rate of the degradation of sensitive compounds [69]. According to Jyothi et al. [70], the diverse particle sizes of powders are due to the wall materials and methods being used in the drying process. The particle size of freeze- and spray-dried powders was greater than 50 *μ*m (Table 3), which can be classified as microparticles or microcapsules. Meanwhile, for both drying methods, the powders encapsulated with CAP and HICAP possessed the lowest bulk density among others. This finding is consistent with those of Porrarud and Pranee [71], which showed that Zn-chlorophyll derivative powder from pandanus (or *pandan* in Malay) has a low bulk density value with a small particle size value. Nonetheless, powder with low bulk density occludes more air within it, and hence, the possibility of oxidative degradation and reduced storage stability is high [32]. As shown in Table 3, encapsulation with different wall materials and drying methods had no significant effect on the powder's cohesiveness and flowability, which all of them can be classified as having intermediate cohesiveness (HR 1.2–1.4) with fair flowability (CI 20–35) [72]. As stated by Çalışkan Koç and Dirim [72], a high HR of the powder indicates that the powder is more cohesive with restricted flowability.

The effects of wall materials on the microencapsulation (spray- and freeze-drying) methods were also evaluated based on the antioxidant activity and retention of TZCD of Zn-amaranth powder. As shown in Table 4, the spray-dried powder encapsulated with GA exhibited the highest antioxidant activity. This finding is in accordance with that of Horszwald et al. [27], which reported that spray-dried aronia powder had higher antioxidant activity than that of freeze-dried. A plausible explanation could be the shorter drying time of the spray-drying process; hence, the rate of thermal degradation of bioactive compounds was considerably lower [73].

In addition, the loss in bioactivity could be associated with material grinding after the drying process in freeze-drying. The grinding process increased the possibility of contact between the powders and the air, resulting in an oxidation reaction leading to the deterioration of the active ingredients [50]. On contrary, the freeze-dried powder demonstrated higher retention of TZCD than spray-dried powder. These findings were comparable to those obtained by Ravichandran et al. [25], Wilkowska et al. [26], and Oberoi and Sogi [74] in the recovery of betalain in beetroots, anthocyanins in blueberry, and lycopene in watermelon, respectively.

**Figure 5:**
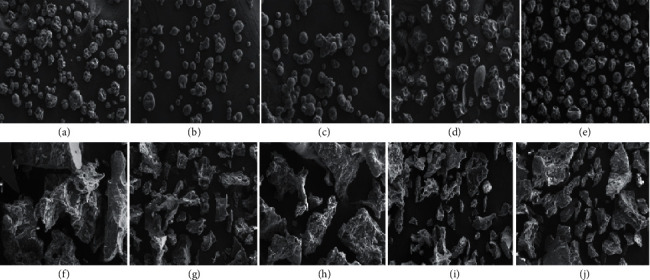
Surface morphology (×200 magnification) of (a–e) spray- and (f–j) freeze-dried Zn-amaranth powders encapsulated with (a, f) MD, (b, g) RMD, (c, h) HICAP, (d, i) CAP, and (e, j) GA (MD: maltodextrin; RMD: resistant maltodextrin; HICAP: HI CAP 100; CAP: Capsul; GA: gum Arabic).

As demonstrated in Figure 5, different drying processes and types of wall material resulted in powders with different particle morphologies. The spray-dried Zn-amaranth powders produced by MD, CAP, and GA displayed irregularly spherical shaped particles with many shrinkages and dents on the surfaces. Saénz et al. [75] reported that these dents are formed during drying and cooling. Additionally, a high inlet temperature during spray-drying accelerated the powder drying rate, resulting in microcapsules of varying sizes and shrinkage [76]. The presence of these dents and shrinkage negatively impacts the flow properties of powder particles. Meanwhile, on the surface of the particles, no visible hole, fracture, crack, and agglomeration were observed that could lead to the deterioration and oxidation of the exposed encapsulated material [77], which is a good indicator of the microencapsulation efficiency of the preparation process of microcapsules *via* spray-drying [78]. Furthermore, comparable SEM images were obtained by spray-drying of fruit or vegetable juices with maltodextrin, which also demonstrated a spherical shape with numerous dents [79–81]. This has been reported to be a typical characteristic of powder obtained by the spray-drying process [61, 82].

Meanwhile, spray-dried powders produced by RMD and HICAP exhibited a spherical shape with a smooth surface. As reported by Osorio et al. [83], smooth spheres are required for the stability of encapsulated ingredients. According to Tonon [10], particles with a rough surface have more contact area than those with a smooth surface, making them more vulnerable to degradation and oxidation. The SEM morphology of both powders was similar to that reported by Ramakrishnan et al. [84] during the spray-drying of tamarillo powder with RMD, as well as in spray-dried carrot powders with RMD and HICAP observed by Shaaruddin et al. [85]. The powders obtained by freeze-drying, however, exhibited an irregular shape and slab-like structure and were substantially larger than the microparticles obtained by spray-drying. Moreover, the freeze-dried microparticles also resembled a broken glass structure, which is a typical feature of freeze-dried powder and that was in line with the results reported by Franceschinis et al. [54], Chávez and Ledeboer [50], and Rocha-Parra et al. [86].

## 4. Conclusions

The findings of this study indicated that the enzymatic liquefaction process, encapsulation technique (spray- and freeze-drying), and wall material (MD, RMD, HICAP, CAP, and GA) substantially influenced the physicochemical properties and morphology of the Zn-amaranth microcapsules. The liquefaction process with Viscozyme L (1% v/w) at pH 5, 45°C for 3 h of incubation, significantly reduced the viscosity and highly retained the TZCD of the Zn-amaranth puree. In comparison between the two encapsulation techniques, freeze-dried powders exhibited better properties in terms of yield, hygroscopicity, and total Zn-chlorophyll derivative retention. As for the wall materials, among the five that were studied, Zn-amaranth powders with maltodextrin and resistant maltodextrin produced the highest yield through freeze-drying. Nevertheless, the high operating cost of freeze-drying should be considered to determine the feasibility of this method. Undoubtedly, the spray-dried powders also possessed appreciable quality due to their lower moisture content, higher encapsulation efficiency, and higher antioxidant activity compared to the freeze-dried powders. In comparison to the other wall materials, spray-dried powder with GA had a higher antioxidant content. In general, all powders showed an intermediate cohesiveness with fair flowability. Overall, our studies have provided a comprehensive understanding of the encapsulation techniques and wall materials to be applied in the manufacture of amaranth powder.

## Figures and Tables

**Table 1:** Enzymes employed in the experiment^a^.

Enzyme	Main activity	Source	The optimal condition of the main activity	
			pH	Temperature (°C)
Viscozyme L	Cellulase, hemicellulase, pectinase, arabanase, *β*-glucanase, and xylanase	*Aspergillus niger*	3.3–5.5	25–55
Pectinex Ultra SP-L	Pectinase and hemicellulase	*Aspergillus aculeatus*	3.5–6.0	30–50
Rapidase PAC	Pectinase and cellulase	*Aspergillus niger*	4.0–5.0	10–55

^a^Information obtained from manufacturer's product sheets.

**Table 2:** Properties of spray- and freeze-dried Zn-amaranth powders encapsulated with 10% (w/w) of different wall materials.

Drying method	Wall material	Process yield (%)	Moisture content (%)	Water activity, *a*_*w*_	Hygroscopicity (%)	Encapsulation efficiency (%)
Spray-drying	MD	65.05 ± 1.04^d^	4.19 ± 0.13^a^	0.48 ± 0.04^a^	12.70 ± 0.53^b^	91.78 ± 0.47^a^
	RMD	72.49 ± 1.04^c^	4.62 ± 1.09^a^	0.45 ± 0.01^a^	15.83 ± 0.46^ab^	84.65 ± 0.42^c^
	HICAP	88.38 ± 0.20^a^	4.05 ± 0.10^a^	0.40 ± 0.01^b^	16.70 ± 1.83^a^	89.69 ± 0.88^b^
	CAP	64.72 ± 0.79^d^	3.52 ± 0.15^a^	0.49 ± 0.02^a^	12.51 ± 0.36^b^	90.58 ± 0.27^ab^
	GA	80.20 ± 0.57^b^	4.47 ± 0.15^a^	0.51 ± 0.01^a^	15.53 ± 0.06^ab^	90.44 ± 1.11^ab^
Freeze-drying	MD	96.95 ± 1.37^a^	5.69 ± 0.55^b^	0.48 ± 0.04^a^	7.52 ± 0.16^c^	87.13 ± 0.30^a^
	RMD	93.94 ± 1.87^a^	4.62 ± 0.99^b^	0.45 ± 0.01^a^	9.69 ± 0.05^b^	86.30 ± 0.34^b^
	HICAP	66.35 ± 0.39^d^	5.99 ± 0.22^b^	0.40 ± 0.01^b^	10.84 ± 0.04^a^	85.59 ± 1.30^b^
	CAP	86.30 ± 0.38^b^	4.93 ± 0.52^b^	0.49 ± 0.02^a^	7.06 ± 0.01^c^	88.56 ± 1.25^a^
	GA	83.01 ± 1.03^c^	8.38 ± 0.14^a^	0.51 ± 0.01^a^	7.18 ± 0.21^c^	82.49 ± 0.15^c^

Data are expressed as the means ± standard deviations of triplicates. Means with the same letter in each column mean not significantly different (*p* < 0.05, Tukey test). MD: maltodextrin; RMD: resistant maltodextrin; HICAP: HI CAP 100; CAP: Capsul; GA: gum Arabic.

**Table 3:** Powder densities with Hausner ratio and Carr index values of spray- and freeze-dried Zn-amaranth powders encapsulated with 10% (w/w) of different wall materials.

Drying	Wall material	Particle size (*μ*m)	Bulk density (g/mL)	Hausner ratio, HR	Carr's index, CI (%)
Spray-drying	MD	64.49 ± 1.82^c^	0.57 ± 0.02^bc^	1.29 ± 0.09^a^	29.49 ± 8.55^a^
	RMD	94.65 ± 3.66^a^	0.68 ± 0.03^a^	1.19 ± 0.10^a^	21.79 ± 5.55^a^
	HICAP	61.18 ± 1.18^d^	0.49 ± 0.40^cd^	1.27 ± 0.10^a^	27.27 ± 9.94^a^
	CAP	70.34 ± 4.03^b^	0.44 ± 0.02^d^	1.24 ± 0.02^a^	23.59 ± 2.36^a^
	GA	60.67 ± 1.15^d^	0.62 ± 0.06^ab^	1.22 ± 0.07^a^	22.34 ± 6.62^a^
Freeze-drying	MD	241.92 ± 2.69^d^	0.36 ± 0.02^b^	1.22 ± 0.08^a^	22.13 ± 7.90^a^
	RMD	292.27 ± 2.73^a^	0.37 ± 0.01^b^	1.21 ± 0.05^a^	20.95 ± 5.49^a^
	HICAP	285.02 ± 2.81^b^	0.38 ± 0.02^b^	1.19 ± 0.02^a^	19.37 ± 2.05^a^
	CAP	252.08 ± 2.56^c^	0.34 ± 0.02^b^	1.21 ± 0.04^a^	20.50 ± 3.68^a^
	GA	172.12 ± 2.58^e^	0.52 ± 0.02^a^	1.16 ± 0.04^a^	16.20 ± 4.41^a^

Data are expressed as the means ± standard deviations of triplicates. Means with the same letter in each column mean not significantly different (*p* < 0.05, Tukey test). MD: maltodextrin; RMD: resistant maltodextrin; HICAP: HI CAP 100; CAP: Capsul; GA: gum Arabic.

**Table 4:** Antioxidant activity and TZCD of spray- and freeze-dried Zn-amaranth powders encapsulated with 10% (w/w) of different wall materials.

Drying method	Wall material	Antioxidant activity (mM (TE)/g DW)		TZCD (%)
		FRAP	DPPH	
Spray-drying	MD	13.24 ± 0.66^c^	8.73 ± 0.05^e^	37.70 ± 1.81^b^
	RMD	14.17 ± 0.23^c^	9.71 ± 0.08^c^	34.13 ± 1.01^c^
	HICAP	15.68 ± 0.41^b^	8.99 ± 0.03^d^	46.65 ± 0.85^a^
	CAP	16.90 ± 0.81^b^	10.10 ± 0.14^b^	35.29 ± 1.08^bc^
	GA	22.04 ± 0.05^a^	12.26 ± 0.03^a^	36.21 ± 0.19^bc^
Freeze-drying	MD	6.48 ± 0.19^c^	7.50 ± 0.18^d^	91.23 ± 1.14^b^
	RMD	6.38 ± 0.12^c^	8.11 ± 0.04^c^	96.95 ± 1.10^a^
	HICAP	7.00 ± 0.14^bc^	8.23 ± 0.08^c^	98.96 ± 0.28^a^
	CAP	7.33 ± 0.55^b^	8.60 ± 0.07^b^	90.81 ± 0.75^b^
	GA	9.04 ± 0.15^a^	10.10 ± 0.08^a^	91.10 ± 1.32^b^

Data are expressed as the means ± standard deviations of triplicates. Means with the same letter in each column mean not significantly different (*p* < 0.05, Tukey test) (MD: maltodextrin; RMD: resistant maltodextrin; HICAP: HI CAP 100; CAP: Capsul; GA: gum Arabic).

## Data Availability

The data used to support the findings of this study are available from the corresponding author upon request.
